# Lipid metabolic dysregulation-induced neuroinflammation in the pathophysiology of major depressive disorder

**DOI:** 10.3389/fimmu.2025.1625087

**Published:** 2025-09-26

**Authors:** Ziyu Ge, Yongdong Hu, Weijing Kan, Lei Li, Jiyi Xu, Yi Zhang, Nan Zheng, Gang Wang, Jing Du

**Affiliations:** ^1^ The National Clinical Research Center for Mental Disorders and Beijing Key Laboratory of Mental Disorders, Beijing Anding Hospital, Capital Medical University, Beijing, China; ^2^ Advanced Innovation Center for Human Brain Protection, Capital Medical University, Beijing, China; ^3^ Department of Clinical Psychology, Beijing Chao-Yang Hospital, Capital Medical University, Beijing, China

**Keywords:** lipid dysregulation, depression, neuroinflammation, insulin resistance, leptin, adiponectin, mitochondrial dysfunction, endoplasmic reticulum stress

## Abstract

Depressive disorders exhibit significant comorbidity with lipid dysregulation. Clinical observations indicate that poor lifestyle factors contribute to lipid dysregulation in depressed patients. This dysregulation disrupts gut microbiota homeostasis and endocrine balance. Mitochondria and endoplasmic reticulum, critical organelles for lipid metabolism, also show impaired homeostasis in depression, further contributing to lipid dysregulation. Such alterations activate peripheral and central immune-inflammatory responses, compromise blood-brain barrier integrity, and disrupt neuroimmune cytokine signaling. This process induces and aggravates neuroinflammation, thereby contributing to the onset and progression of depressive disorders. These disruptions in homeostasis further exacerbate lipid dysregulation. This review delineates the molecular mechanisms by which dysregulation of lipid metabolism exacerbates depressive disorders via neuroinflammatory pathways, offering critical insights into pathogenesis and therapeutic strategies.

## 1 Introduction

Major depressive disorder (MDD) is a prevalent mental illness with substantial heterogeneity, affecting 4.4% of the global population (1). While its exact pathogenesis remains unclear, emerging evidence highlights a bidirectional relationship between depression and lipid dysregulation (2). Epidemiological data reveal a 55% increased risk of depression in individuals with obesity and a 58% higher risk of obesity in those with depression (3). Classical depression often presents with appetite loss, whereas atypical depression is characterized by lethargy, weight gain, and cravings for high-calorie foods, frequently accompanied by imbalances in lipid metabolism (4). These lipid metabolism abnormalities in atypical depression are also closely associated with elevated inflammatory markers, prolonged disease course, and reduced treatment response (5).

**Figure 1:**
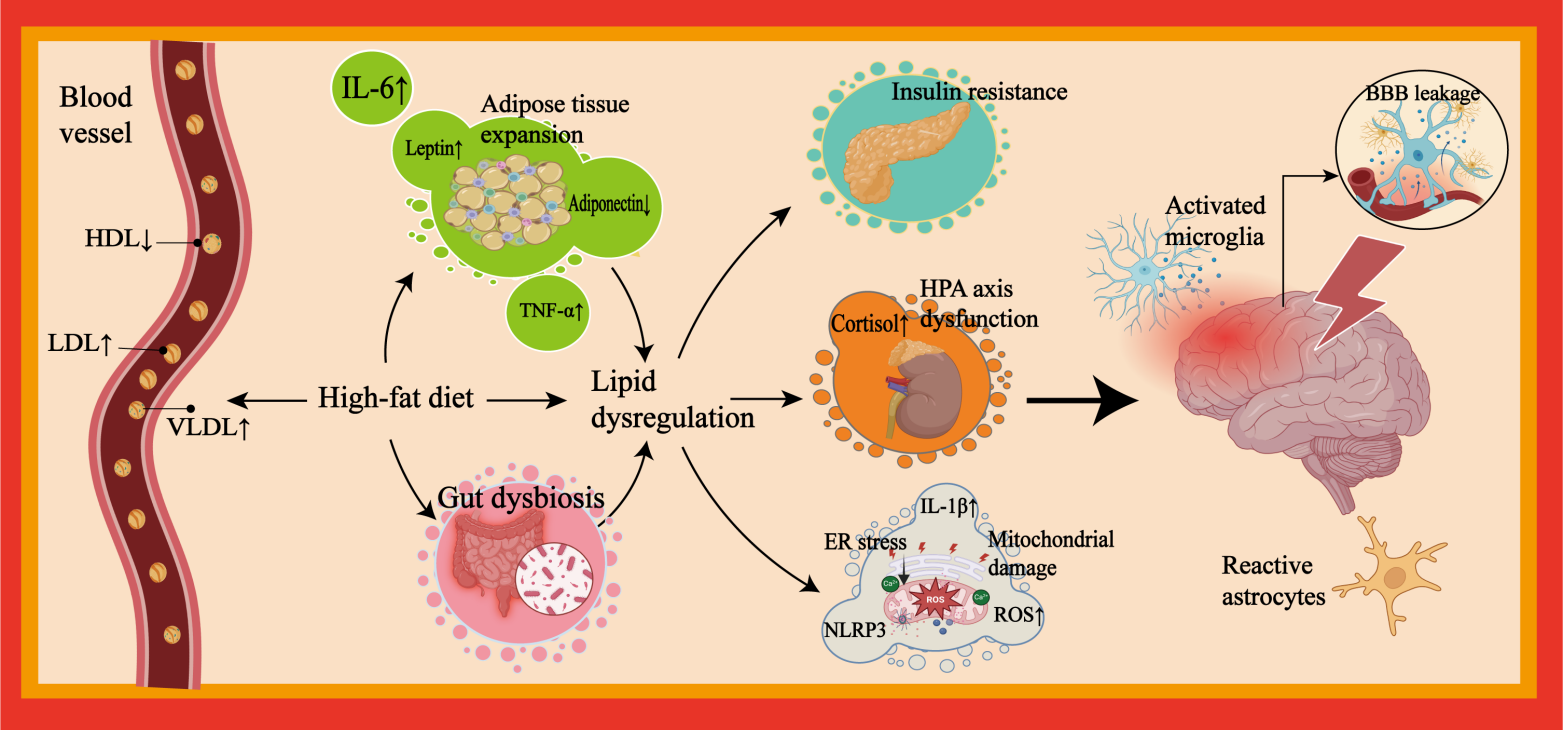
Pathological mechanisms linking lipid dysregulation to neuroinflammation in depression. This figure outlines mechanisms linking lipid dysregulation to neuroinflammation and depression. Depression patients frequently exhibit unhealthy lifestyle habits. Excessive consumption of high-fat foods can induce lipid dysregulation. Their peripheral blood is often characterized by a metabolic imbalance featuring elevated LDL and VLDL levels and reduced HDL levels. Visceral adipose tissue expansion induced by unhealthy dietary patterns is particularly susceptible to immune cell infiltration and cytokine secretion (e.g., IL-6, TNF-α). Furthermore, aided by concomitant gut microbiota dysbiosis resulting from such patterns, a chronic systemic inflammatory state develops. This state promotes and/or accompanies metabolic and endocrine risks (e.g., insulin resistance, HPA axis dysregulation (excessive cortisol secretion), and adipokine dysregulation) and impairment of organelle function (e.g., mitochondrial damage and ER stress). Collectively, these factors exacerbate lipid dysregulation and sustain peripheral inflammation. Persistent peripheral inflammation can lead to compromised BBB integrity, promote neuroinflammation, and induce alterations in neuroplasticity within mood-regulating neural networks, which may ultimately contribute to depressive symptoms. LDL, low-density lipoprotein; VLDL, very low-density lipoprotein; HDL, high-density lipoprotein; IL-6, interleukin-6; TNF-α, tumor necrosis factor-alpha; HPA, hypothalamic-pituitary-adrenal; ER, endoplasmic reticulum; BBB, blood-brain barrier.

Lipids, as critical components for cellular structure and neural function, play vital roles in maintaining membrane integrity, regulating inflammation, and supporting synaptic plasticity (6). Currently, the interplay between lipid dysregulation and neuroinflammation is recognized as a core pathological mechanism in MDD (7–9). Chronic lipid dysregulation-driven peripheral inflammation compromises the integrity of the blood brain barrier (BBB), enabling inflammatory mediators to infiltrate the central nervous system (CNS) (10, 11) (see **Figure 1**). Within the brain, lipid dysregulation activates microglia (6) and disrupts astrocytic lipid homeostasis (12, 13), creating a self-perpetuating cycle of neuroinflammation. These processes impair neurotransmitter metabolism, neural network remodeling, and limbic system function, critical pathways for mood regulation (12, 14, 15). Furthermore, oxidative stress-induced lipid peroxidation modifies cerebral lipid composition and signaling molecules (16), further exacerbating depressive pathophysiology.

## 2 Method

**Figure 2:**
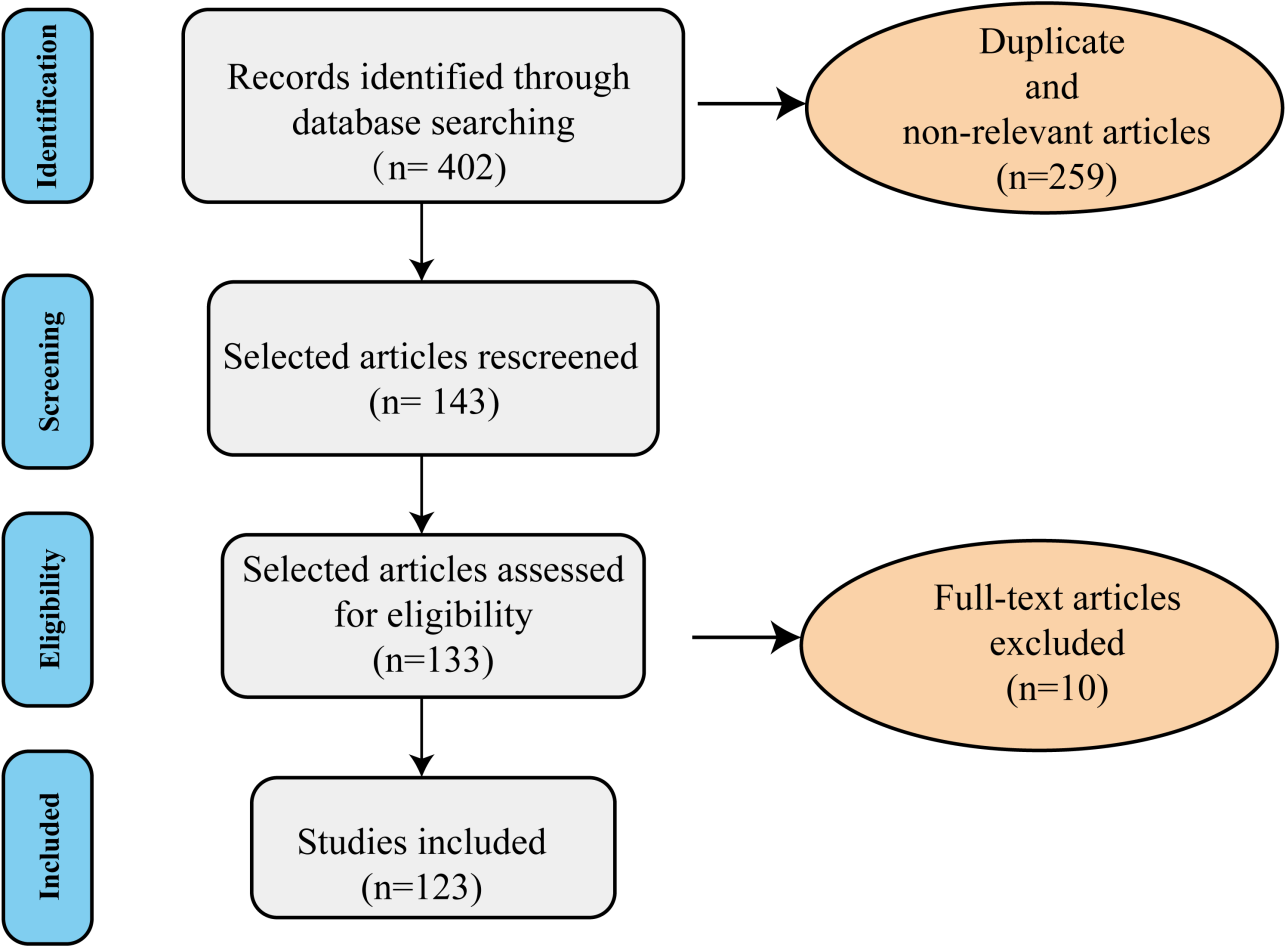
The diagram of the search strategy. The flow chart of review shows the included and excluded studies (duplicated, review articles, non-relevant, and those in non-English language). A total of 123 studies were included in the current literature.

In this comprehensive literature review, to find literature on the role of lipid metabolic dysregulation-induced neuroinflammation in the pathophysiology of major depressive disorder. We searched PubMed and Google Scholar as electronic databases using the combination of the terms ‘lipid metabolism’, ‘inflammation’, and ‘depression’ with the following keywords: lipid dysregulation, obesity, MDD, neuroinflammation, omega-3, cholesterol, gene, gut microbiota, adipose tissue, BBB, Hypothalamic-pituitary-adrenal (HPA), insulin resistance, leptin, adiponectin, brain, and CNS, mitochondrial dysfunction, endoplasmic reticulum stress (ER stress). We included preclinical and clinical studies to provide a comprehensive review article. No time limit (up to 16 July 2025) was considered in this review. We excluded publications in non-English, conference abstracts, review articles, and dissertations. The search strategy used for the current review follows the PRISMA 2009 checklist and is shown in **Figure 2**. In the primary search, 402 studies were obtained. Next, duplicate and non-relevant articles were deleted. Screening of the remaining research was performed by reading the titles, abstracts, or full texts based on the inclusion criteria. Following full-text assessment, 10 articles were excluded for the following reasons: non-relevant disease models (n=4), lack of mechanistic integration (n=4), insufficient data (n=2). Finally, a total of 123 studies were included in the review process. Among the 123 included studies, there are 75 preclinical studies and 48 clinical studies. The results of the included studies in **Tables 1** and **2**.

**Table 1:** Preclinical evidence of the lipid metabolism involved in depression in animal models.

Research topic	Model/intervention	Main findings	References
HFD induces neuroinflammation and depressive-like behaviors in mice	Mice fed HFD	A chronic high-cholesterol diet induces anxiety and depressive-like behaviors in mice.	(68, 69)
		High cholesterol intake specifically activates the TLR4 signaling pathway in the PFC and liver, boosting inflammatory responses in the peripheral and CNS.	
		Downregulation of tight junction proteins (ZO-1, claudin-5) compromises BBB integrity, facilitating the entry of peripheral inflammatory factors into the brain.	(90, 91)
Abnormalities in the Lipid metabolism-related gene	Depression model mice	Aberrant expression of cholesterol metabolism genes (HMGCR, CYP46A1, ABCA1, APOE) in the prefrontal cortex, accompanied by reduced levels of synaptic proteins.	(33)
	Mice with forebrain-specific overexpression	SMPD1 (which generates Cer) overexpression induces depression-like behaviors and promotes neuroinflammation.	(25, 37)
Anti-inflammatory mechanisms of ω-3 PUFAs	CUMS depression model and ω-3 PUFA supplementation	ω-3 PUFAs replace AA in cell membranes, reducing pro-inflammatory mediators; promote the generation of SPMs; inhibit the TLR4/NF-κB pathway in microglia.	(45, 60, 96)
		Chronic HFD feeding in mice significantly increases SFA levels while reducing PUFA content in the brain.	
	Microglia	Additionally, excessive accumulation of SFA can directly activate pattern recognition receptors on immune cells, particularly macrophages.	(55, 56)
High-fat diet disrupts gut microbiota balance	Mice fed HFD	FMT from HFD-fed mice to antibiotic-treated normal-diet recipients increases anxiety-like behaviors, intestinal inflammation, barrier permeability, plasma endotoxin levels, and TLR2/4 expression in the PFC.	(2, 87)
		HFD feeding and obesity induced a shift in the microbiota from Bacteroides to Firmicutes in mice, and the abundance of certain Firmicutes species was positively correlated with energy intake and plasma CRP levels.	
Hypothalamic inflammation and lipid metabolism dysregulation	Obese model mice	SFA accumulation activates the IKKβ/NF-κB pathway in hypothalamic astrocytes, triggering neuroinflammation and metabolic dysfunction.	(98, 101)
	Mice fed HFD	Acute HFD exposure (1 day) elevates hypothalamic IL-6, TNF-α, and microglial activation, with prolonged HFD (3 days) exacerbating neuroinflammation and gliosis.	(103, 104)
		After 1–4 weeks of HFD feeding, astrocytes in hypothalamic regions secrete inflammatory cytokines that amplify inflammatory cascades.	
Mitochondrial dysfunction	HFD-induced obese mice	Mitochondrial β-oxidation overload, ROS accumulation, reduced CL levels, and inhibition of mitophagy.	(159, 161)
Anti-inflammatory role of adiponectin	Chronically stressed mice	Adiponectin/AdipoR1 signaling activates the AMPK pathway, promoting microglial polarization towards the anti-inflammatory M2 phenotype and alleviating neuroinflammation.	(157)

HFD, high-fat diet; TLR4/2, toll-like receptor 4/2; PFC, prefrontal cortex; CNS, central nervous system; BBB, blood-brain barrier; Cer, ceramide; CUMS, chronic unpredictable mild stress; ω-3 PUFAs, omega-3 polyunsaturated fatty acids; AA, arachidonic acid; NF-κB, nuclear factor kappa-light-chain-enhancer of activated B cells; SPMs, specialized pro-resolving mediators; SFA, saturated fatty acid; FMT, fecal microbiota transplantation; CRP, c-reactive protein; IKKβ, IκB kinase beta; IL-6, interleukin-6; TNF-α, tumor necrosis factor-alpha; ROS, reactive oxygen species; CL, cardiolipin; AdipoR1, adiponectin receptor 1; AMPK, AMP-activated protein kinase; MDD, major depressive disorder.

**Table 2:** Clinical Studies on the involvement of lipid metabolism in MDD.

Research topic	Model/intervention	Main findings	References
Depression patients and lipid metabolism dysregulation	Patients with atypical depression	Patients with atypical depression tend high-sugar, high-fat dietary patterns and frequently display dysregulated lipid metabolism.	(62, 63)
Abnormal lipid profiles	MDD patients vs. Healthy controls	Serum: Elevated TG↑, reduced HDL-C↓, elevated LDL-C↑, increased LDL-C/HDL-C. ratio↑. Phospholipid levels (LPC, LPE, PC, PE, PI) positively correlate with depression severity.	(17, 20, 23)
Cer levels and depression severity	Plasma from MDD patients	Increased Cer levels positively correlate with HAMD-D scores. Excessive ceramide elevation can promote inflammation and neurotoxicity.	(24, 28)
Expression of lipid metabolism-related genes	GWAS databases	Genes associated with obesity are also expressed in brain regions involved in mood regulation.	(30, 31)
rTMS modulation of lipid metabolism	Treatment-resistant MDD patients	rTMS applied to the visual cortex for 5 days increased plasma ApoA1↑ and activated the ABCA1 signaling pathway.	(36)
Dietary structure impacts lipid metabolism balance	Cohort of depression patients	A high-sugar, high-fat diet in depressed patients decreases plasma EPA, DHA, and total ω-3 PUFA levels, while increasing SFAs↑. The ω-6/ω-3 PUFA ratio is typically disrupted. Reduced ω-3 PUFA levels promote neuroinflammation.	(43, 44)
Gut microbiota dysbiosis	Depressed/Anxious patients vs. Controls	Obese patients frequently exhibit gut microbiota dysbiosis. Elevated plasma levels of LPS, FABP2, and zonulin (markers of intestinal permeability) correlate with the degree of inflammation.	(85)
Insulin resistance	MDD patients	MDD patients often present with insulin resistance. In obesity, reduced brain insulin sensitivity positively correlates with the severity of depression and anxiety.	(128)
Leptin resistance	Obese MDD patients	Elevated serum leptin levels with impaired leptin receptor function, positively correlating with hyperactivity of the HPA axis and insulin resistance.	(148)
Adiponectin levels	Male depression cohort	Low adiponectin expression significantly increases the risk of MDD.	(154)
Mitochondrial DNA damage	Prefrontal cortex tissue from MDD patients	The extent of mtDNA mutations and oxidative damage in the prefrontal cortex positively correlates with depression symptom severity. MDD patients with lipid dysregulation exhibit reduced CL levels and mitochondrial dysfunction.	(163, 170)

Symbols: ↑ = increased, ↓ = decreased. Arrows indicate the direction of change relative to controls.

HFD, high-fat diet; TLR4/2, toll-like receptor 4/2; PFC, prefrontal cortex; CNS, central nervous system; BBB, blood-brain barrier; Cer, ceramide; CUMS, chronic unpredictable mild stress; ω-3 PUFAs, omega-3 polyunsaturated fatty acids; AA, arachidonic acid; NF-κB, nuclear factor kappa-light-chain-enhancer of activated B cells; SPMs, specialized pro-resolving mediators; SFA, saturated fatty acid; FMT, fecal microbiota transplantation; CRP, c-reactive protein; IKKβ, IκB kinase beta; IL-6, interleukin-6; TNF-α, tumor necrosis factor-alpha; ROS, reactive oxygen species; CL, cardiolipin; AdipoR1, adiponectin receptor 1; AMPK, AMP-activated protein kinase; MDD, major depressive disorder; TG, triglycerides; HDL-C, high-density lipoprotein cholesterol; LDL-C, low-density lipoprotein cholesterol; PC, phosphatidylcholine; LPC, lysophosphatidylcholine; PE, phosphatidylethanolamine; LPE, lysophosphatidylethanolamine; PI, phosphatidylinositol; HAMD-D, Hamilton Depression Rating Scale; NLRP3, NOD-like receptor family pyrin domain containing 3; rTMS, repetitive transcranial magnetic stimulation; EPA, eicosapentaenoic acid; DHA, docosahexaenoic acid; ω-6 PUFAs, omega-6 polyunsaturated fatty acids; LPS, lipopolysaccharide; FABP2, fatty acid-binding protein 2; HPA, hypothalamic-pituitary-adrenal; mtDNA, mitochondrial DNA.

(Human Evidence).

## 3 Lipid dysregulation and inflammatory mechanisms in MDD

### 3.1 Lipid profile alterations: clinical features and pathological relevance

Depressed patients frequently exhibit pro-inflammatory lipid metabolic features, as outlined below:

(1) Lipoprotein Abnormalities: Patients demonstrate elevated serum triglyceride (TG) levels, decreased high-density lipoprotein cholesterol (HDL-C), and significantly increased low-density lipoprotein cholesterol (LDL-C) along with an elevated LDL-C/HDL-C ratio. These alterations reflect abnormal lipid metabolism and are closely associated with the presence, severity, and early stages of depressive symptoms (17, 18).

It is hypothesized that an elevated TG/HDL ratio may signal increased free fatty acid (FFA) release. The increase in FFA can promote the secretion of pro-inflammatory cytokines such as interleukin-6 (IL-6) and tumor necrosis factor-alpha (TNF-α) (19).

(2) Phospholipid Metabolism Imbalance: As depressive symptoms worsen, serum levels of lysophospholipids (lysophosphatidylcholine [LPC], lysophosphatidylethanolamine [LPE]) significantly increase, while levels of alkyl phosphatidylethanolamine (O-alkyl phosphatidylethanolamine [PE-O]), which possesses antioxidant function, decrease (20). LPC promotes monocyte migration and increases the production of pro-inflammatory cytokines by activating endothelial G protein-coupled receptors (GPCRs) (21). Furthermore, LPC contributes to demyelination, cognitive deficits, and microglia-mediated neuroinflammation (22). Compared with healthy individuals, plasma levels of phosphatidylcholine (PC), phosphatidylethanolamine (PE), and phosphatidylinositol (PI) are also significantly increased in depressed patients, with levels increasing in proportion to symptom severity (20, 23).

(3) Sphingolipid Metabolism Abnormalities: Plasma ceramide (Cer) levels are also significantly increased in depressed patients compared to healthy controls and correlate with the severity of the Hamilton Depression Rating Scale (HAMD-D) (24). Under depressive conditions, oxidative stress activates acid sphingomyelinase (ASM), leading to the catabolism of sphingolipids into Cer (25). Increased ASM and Cer levels inhibit the effects of antidepressant medications (26, 27). Excessive Cer accumulation not only activates the NOD-like receptor family pyrin domain containing 3 (NLRP3) inflammasome, triggering the release of numerous inflammatory factors (28), but also forms pro-apoptotic channels in the mitochondrial membrane. This damages the electron transport chain, leading to increased reactive oxygen species (ROS) and decreased ATP production (29). These changes are both pro-inflammatory and neurotoxic, thereby exacerbating the pathological progression of depression **Table 3** compares the lipid dysregulation profiles between depression patients and preclinical models..

**Table 3:** Comparative lipid dysregulation profiles in depression: Clinical and preclinical evidence.

Lipid class	Key biomarkers	Human clinical evidence	Preclinical evidence (models)	Sample	References
Sphingolipid	Cer(C18:0) ↑ Cer(C20:0) ↑	Increased in MDD Plasma, correlates with the severity of HAMD-D.	Increase in the hippocampus and PFC of CUMS mice and rats. Inhibits the proliferation of hippocampal neurons *in vitro*. Reversed by antidepressant drugs.	Plasma, Brain, Hippocampal neurons.	(24, 27, 196–199)
	SM 26:1 ↓ SM 39:2 ↓	SM23:1/SM16:0 ratio was negatively correlated with the severity of depression symptoms			
Phospholipid	PE↑ PC↑ LPE↑ LPC↑	LPC, LPE, PC, PE, PI increased with the increase in symptom severity. PE-O decreased with the rise of symptom severity	Dysregulated PE metabolism in depressed mice. Reduced PE and elevated LPE levels were observed in the prefrontal cortex of CUMS rats.	Plasma, Brain.	(20, 23, 24, 196, 200, 201)
ω-3 PUFAs	EPA ↓ DHA ↓ EPA/AA ↓	Patients with depression exhibited significantly lower levels of EPA, DHA, and total ω-3 PUFAs compared to controls.	In depressed rat, EPA effectively ameliorated depressive-like behaviors, glial dysfunction, and hippocampal apoptotic signaling.	Plasma, Brain, hippocampal progenitor cells.	(45, 202–206)
		Higher AA to (EPA+DHA) ratios showed positive correlations with increasing MADRS scores over time and elevated IL-6 levels.	ω-3 PUFAs prevent inflammatory damage to human hippocampal progenitor cells.		
		Elevating EPA and DHA concentrations significantly reduced depressive symptoms.			
Triglycerides	VLDL-TG ↑	TG increased with increase of symptom severity. Depressed patients demonstrate elevated serum lipid profiles, particularly higher TG levels, compared to healthy controls.	Increased serum in HFD models.	Serum, Brain.	(17, 207–209)

Symbols: ↑ = increased, ↓ = decreased. Summary of consistent lipid alterations in depression. Arrows indicate the direction of change relative to controls.

Cer, ceramide; SM, sphingomyelin; MDD, major depressive disorder; HAMD-D, Hamilton Depression Rating Scale; CUMS, chronic unpredictable mild stress; PFC, prefrontal cortex; PC, phosphatidylcholine; LPC, lysophosphatidylcholine; PE, phosphatidylethanolamine; LPE, lysophosphatidylethanolamine; PI, phosphatidylinositol; PE-O, plasmalogen (ether-linked PE); EPA, eicosapentaenoic acid; DHA, docosahexaenoic acid; AA, arachidonic acid; ω-3 pufas, omega-3 polyunsaturated fatty acids; VLDL-TG, very low-density lipoprotein triglyceride; HFD, high-fat diet.

### 3.2 Lipid metabolism-related gene expression abnormalities in MDD

Lipid profile abnormalities in depressed patients not only reflect metabolic disorders but also may be directly involved in the pathological process of depression through lipid-mediated inflammatory signaling pathways. Recent studies have revealed that such metabolic disorders may be closely related to the abnormal expression of key genes in lipid metabolism, thus forming a “gene-metabolism-inflammation” cascade regulatory network.

Studies of obesity-related genes revealed that genome-wide association analysis (GWAS) screened several genes highly associated with body mass index (BMI) and obesity status (30, 31). These genes were highly expressed in key brain regions regulating appetite and energy homeostasis (e.g., hypothalamus and pituitary gland), as well as mood-regulating brain regions (e.g., hippocampus and limbic system) (32). Depression model mice were found to have significantly abnormal expression of cholesterol metabolism-related genes (e.g., *HMGCR*, *CYP46A1*, *ABCA1*, *APOE*, etc.) in the prefrontal region, accompanied by a decrease in the synaptic proteins PSD-95 and synaptophysin, suggesting that an imbalance in cholesterol metabolism not only affects synaptic plasticity in neurons but also directly induces depressive behaviors (33). As a cholesterol transporter, common variants of *ABCA1* decrease HDL levels, exacerbate oxidative stress, and damage neurons and glial cells (34).

Research examining cytokine modulators in monocytes of MDD patients has been conducted to investigate the role of inflammation in depression. The results showed an upregulation of ABCA1 in these cells (35), suggesting increased monocyte activation. Repetitive transcranial magnetic stimulation (rTMS), Food and Drug Administration (FDA) -approved treatment for resistant MDD, was also assessed in another study for its effect on the ABCA1/ApoA1 signaling pathway (36). This pathway involves ABCA1 loading lipids onto apoA1 to form nascent HDL particles (34). Moreover, the study revealed that MDD patients had lower plasma apoA1 levels, a deficit that was reversed following 5 days of rTMS applied to the visual cortex (36).

SMPD1, a type of ASM, which produces ceramide, maintains cell membranes, and regulates cellular functions. It is upregulated in depressed patients. Forebrain-specific overexpression of SMPD1 causes depression-like symptoms in mice (37). Increased activity of ASM is a hallmark of MDD metabolic alterations, which promotes the breakdown of sphingolipids into ceramides, which in turn induces neuroinflammation (25), leading to neuronal apoptosis and impaired synaptic plasticity.

Phospholipase A2 (PLA2) expression is upregulated in depressed patients and hydrolyzes membrane phospholipids to arachidonic acid (AA) and lysophospholipids (38). AA acts as a prostaglandin precursor, generating prostaglandins through the action of cyclooxygenase (COX). When AA levels in cell membrane lipids are too high, inflammatory substances are formed, among which prostaglandin E2 (PGE2) is a potent pro-inflammatory factor (39). It has been found that PGE2 activates microglia, induces them to secrete large amounts of pro-inflammatory factors, enhances the permeability of the BBB, promotes the entry of peripheral immune cells into the CNS, and expands the scope of neuroinflammation (39). Lysophospholipids can be converted into platelet-activating factors, and these lipid mediators play a key role in the initiation, maintenance, and regulation of neuroinflammation and oxidative stress (40). Abnormal expression of phospholipid metabolism genes may lead to altered phospholipid composition, which in turn affects neuronal membrane fluidity and signal transduction function.

### 3.3 Peripheral lipid dysregulation and inflammatory cascades in MDD

#### 3.3.1 Fatty acid metabolism, which regulates inflammation, contributes to the pathogenesis of MDD

Fatty acids serve as key molecules for brain integrity and executive function, but imbalances in their metabolism can significantly contribute to inflammatory responses. Since the human body is unable to synthesize essential fatty acids (EFAs), their mechanism of maintaining physiological homeostasis through dietary supplementation is of great interest (39). Studies have shown that depression is negatively correlated with dietary structure: the Mediterranean diet [high in omega-3 (ω-3) polyunsaturated fatty acids (PUFAs), antioxidants] is associated with lower levels of inflammatory markers, while the Western dietary pattern (rich in high-sugar, high-fat foods) is associated with higher levels of C-reactive protein (CRP) (41). Externally supplemented essential fatty acids are mainly categorized as ω-3 and omega-6 (ω-6). The ω-3 pathway produces anti-inflammatory mediators such as eicosapentaenoic acid (EPA)/docosahexaenoic acid (DHA) (42), while the ω-6 pathway generates arachidonic acid-derived pro-inflammatory mediators, which in turn modulate inflammatory states (39). Patients with severe depression often exhibit lower levels of ω-3 (43), and their ω-6 to ω-3 ratio is typically imbalanced, which may exacerbate inflammatory responses (44).

ω-3 can inhibit neuroinflammation by reducing the production of pro-inflammatory cytokines and enhancing anti-inflammatory signaling. Supplemental ω-3 PUFAs are incorporated into cell membranes, replacing the pro-inflammatory AA within the membrane (45). This substitution reduces the production of inflammatory mediators such as prostaglandins, thromboxanes, and leukotrienes. Furthermore, ω-3 PUFAs mediate the production of specialized pro-resolving mediators (SPMs), including resolvins, protectins, and maresins (46, 47). These mediators actively terminate ongoing inflammatory processes by reducing the infiltration of inflammatory cells, inhibiting the release of pro-inflammatory cytokines, and promoting the clearance of cellular debris (48). ω-3 PUFAs also influence oxidative stress pathways, which are closely linked to inflammation. By modulating the activity of enzymes such as COX-2 (49), ω-3 PUFAs reduce the production of ROS and nitric oxide (NO) (50), both of which contribute to inflammatory tissue damage.

When cells are damaged or stressed, they release damage-associated molecular patterns (DAMPs), such as high mobility group box 1 protein (HMGB1) and S100 proteins (48). These DAMPs serve as danger signals that activate immune cells, leading to an inflammatory response (51, 52). Released DAMPs bind to specific receptors on microglia, such as Toll-like receptors (TLRs), mainly TLR2 and TLR4, and the receptor for advanced glycation end products (RAGE) (53). This binding triggers a series of intracellular signaling pathways, including nuclear factor kappa-light-chain-enhancer of activated B cells (NF-κB) activation (54). Once activated, NF-κB moves to the nucleus, where it promotes the transcription of pro-inflammatory genes, including cytokines, chemokines, and other mediators involved in inflammation, thereby initiating inflammatory pathways (48). Additionally, excessive accumulation of saturated fatty acid (SFA) can directly activate pattern recognition receptors on immune cells (particularly macrophages), such as NF-κB and its downstream signaling pathways, triggering a DAMP-like inflammatory response (45, 55–58). Conversely, ω-3 PUFAs can downregulate the expression of TLRs on immune cells. This reduces the likelihood of interaction between DAMPs and their receptors, consequently suppressing downstream inflammatory pathways (59–61). Supplementation with ω-3 PUFAs reduces inflammatory factor-induced decrease in neurogenesis and increase in apoptosis in human hippocampal progenitor cells, thereby improving symptoms in depressed patients (45).

It is worth noting that lipid dysregulation is prevalent in patients with atypical depression, and their high-sugar, high-fat dietary preferences lead to an elevated serum SFA ratio (62), accompanied by significant depletion of EPA and DHA (63). Several studies have shown that higher ω-3 PUFAs intake is associated with lower prevalence and severity of mood disorders and may reduce neuroinflammation and alleviate mood deficit behaviors in rodents (64–66).

Chronic intake of SFAs induces visceral fat deposition and adipose tissue hypertrophy. When adipose tissue expands beyond its blood supply capacity, it becomes dysfunctional and inflammatory, increasing pro-inflammatory M1 macrophage infiltration and interfering with metabolic processes such as islet signaling (67). Animal studies have shown that a chronic high-cholesterol diet induces anxiety and depressive-like behaviors in mice. High cholesterol intake specifically activates the TLR4 signaling pathway in the PFC and liver, boosting inflammatory responses in the peripheral and CNS (68, 69).

#### 3.3.2 Gut dysbiosis crosstalks to the lipid-regulated neuroinflammation in MDD

Lipid dysregulation not only directly regulates inflammation but also establishes cross-systemic neuroinflammatory networks by reprogramming the gut microbiota through lipid microenvironment disturbances. The diversity and homeostasis of the gut microbiota an important indicators of the health of the organism and have been shown to affect lipid metabolism and lipid levels in blood and tissues of mice and humans (70). A stable gut microbiota is essential for resistance to pathogenic infections and maintenance of immune system homeostasis.

Obese individuals often have gut microbiomes that enhance energy extraction and fat accumulation (71). Inflammation arising from gut dysbiosis induced by lipid dysregulation can impair leptin expression and disrupt thermogenesis, thereby contributing to obesity (72). High-fat diet (HFD) feeding and obesity induced a shift in the microbiota from *Bacteroides* to *Firmicutes* in mice, and the abundance of certain *Firmicutes* species was positively correlated with energy intake and plasma C-reactive protein (CRP) levels (2). The gut microbiota modulates host lipid metabolism and immune responses by producing metabolites, including short-chain fatty acids (SCFAs) and secondary bile acids, as well as pro-inflammatory bacterial-derived factors such as lipopolysaccharides (LPS).

Studies demonstrate that gut microbiota and their metabolites influence the brain through direct vagal signaling or indirect immune-neuroendocrine pathways (73). Specifically, SCFAs stimulate microglia to secrete the neuroprotective cytokine IL-10, while metabolites like indole derivatives regulate microglial activation and neurotoxicity. Furthermore, elevated circulating bile acids (BAs), generated by bacterial metabolism, may disrupt tight junction integrity, enhance BBB permeability, and facilitate the entry of BAs or peripheral cholesterol into the CNS (74). Western diets reduce the production of SCFAs by the gut microbiota, consequently diminishing mucus secretion, antimicrobial protein synthesis, and regulatory T cell (Treg) differentiation. This impairs intestinal barrier integrity and compromises tight junction proteins (75, 76). Furthermore, such diets increase BAs synthesis, which emulsifies and degrades the intestinal mucus layer, thereby elevating gut permeability (77). Increased intestinal permeability allows inflammation-inducing substances-including LPS-to translocate into systemic circulation, instigating peripheral and systemic inflammation (78).

Beyond dietary factors, psychological stress alters gut microbiota profiles in MDD patients and depressive mouse models (79). MDD patients exhibit reduced *Clostridia* abundance, which negatively correlates with anxiety and depression severity. The study also found differences in the composition of the gut microbiota in depressed patients, including those in remission, compared to healthy controls (80), characterized by decreased butyrate-producing anti-inflammatory bacteria and increased pro-inflammatory taxa (81). Additionally, SCFA-producing and anti-inflammatory bacterial populations are diminished in anxiety and depression (82). Research indicates that gut barrier damage triggered by dysbiosis may also be linked to depression (78, 83). Depression patients exhibit higher levels of blood IgM and IgA antibodies binding to commensal gut bacterial LPS (84). A clinical study found significantly elevated plasma levels of LPS, fatty acid binding protein 2 (FABP2), and zonulin (potential markers of gut integrity) in depressed/anxious patients compared to non-depressed/anxious controls (85). Patients with MDD exhibiting suicidal behavior showed lower plasma zonulin and higher FABP levels compared to non-suicidal MDD patients and healthy controls. Furthermore, IL-6 was inversely correlated with zonulin and proportionally correlated with FABP (86). These findings provide further support for the concept that dysbiosis-induced intestinal barrier impairment may be associated with chronic depression.

Fecal microbiota transplantation (FMT) from HFD-fed mice to antibiotic-treated normal-diet recipients increases anxiety-like behaviors, intestinal inflammation, barrier permeability, plasma endotoxin levels, and TLR2/4 expression in the PFC (87). Gut dysbiosis further exacerbates intestinal epithelial barrier dysfunction, promoting pro-inflammatory cytokine release and neuroinflammatory cascades.

#### 3.3.3 The inflammatory transmission from the peripheral to the central nervous system in MDD

Peripheral inflammation induces neuroinflammation by compromising BBB integrity (88). Elevated peripheral inflammatory mediators and lipid dysregulation destabilize the BBB, increasing its permeability. A key mechanism involves disrupted expression of tight junction proteins (e.g., zonula occludens-1 [ZO-1], claudin-5, and occludin) (89). Experimental evidence shows that 8 weeks of HFD feeding in mice significantly reduces ZO-1, claudin-5, and occludin protein levels, causing cerebral microvascular leakage (90). Prolonged HFD exposure (13 weeks) further downregulates tight junction protein mRNA (notably claudin-5 and claudin-12) in the choroid plexus and BBB (91). Circulating cytokines crossing the permeable BBB disrupt depression-associated brain signaling (11, 92), suggesting that lipid dysregulation-induced BBB dysfunction facilitates aberrant peripheral-CNS communication, contributing to inflammation-driven depressive behaviors. Peripherally activated microglia suppress neural stem cell proliferation, enhance neural progenitor cell apoptosis, and impair neurogenesis by reducing newborn neuron survival and synaptic integration (93). Moreover, monocytes, macrophages, and T cells infiltrate the CNS, amplifying neuroinflammation via cytokine secretion.

Beyond BBB disruption, peripheral inflammatory factors or chemokines activate perivascular macrophages through receptors on astrocytes and vascular endothelial cells, generating inflammatory mediators. Lipid dysregulation-driven oxidative stress and cytokines further propagate neuroinflammation via vagal afferent signaling or CNS NF-κB pathway activation (2, 94). Ultimately, peripheral inflammatory signals trigger glial activation and neurotransmitter dysregulation, highlighting the systemic-to-central pathological cascade of lipid dysregulation.

### 3.4 The central lipid dysregulation and neuroinflammatory signaling cascades in the pathogenesis of MDD

The normal functioning of the CNS is highly dependent on the fine-tuning of the lipid network. Neuronal membranes and myelin sheaths are rich in fatty acids and lipids, and these components are essential for their structure and function. Given the limited capacity of the brain to synthesize lipids, it must rely on the supply of lipids from the peripheral circulation. Therefore, any imbalance in lipid metabolism may trigger a systemic inflammatory response that threatens neuronal survival and function. Fatty acid transport activity in the brain is significantly enhanced in patients with metabolic syndrome compared to healthy people (95). Animal studies reveal that chronic HFD feeding in mice significantly increases SFA levels while reducing PUFA content in the brain (96). These alterations critically impair neuronal survival and function.

In the context of CNS inflammation, astrocytes play a critical role alongside microglia. In the depression model mice, astrocytes exhibit impaired mitochondrial function. When fatty acid load exceeds their oxidative phosphorylation capacity, these cells transition into reactive astrocytes (12). Reactive astrocytes not only promote synaptic dysfunction and neuroinflammation but also trigger metabolic reprogramming in neurons and microglial activation, while suppressing fatty acid and phospholipid biosynthesis required for remyelination. This positive feedback mechanism accelerates disease progression (12).

Astrocytes are capable of monitoring and metabolizing lipids; however, excessive lipid accumulation surpasses their homeostatic capacity. Obesity elevates circulating levels of SFAs (97). Under SFA-enriched conditions, hypothalamic astrocytes accumulate lipid droplets. These lipid-laden cells demonstrate increased expression of astrogliosis markers (e.g., GFAP) at mRNA and/or protein levels, promoting ER stress-mediated release of proinflammatory cytokines that induce reactive astrogliosis and microglial activation (98, 99). Reactive astrocytes release GABA to inhibit neurons in the lateral hypothalamic area, leading to increased fat deposition (100) and exacerbation of lipid dysregulation.

Lipid dysregulation activates IκB kinase beta (IKKβ)/NF-κB signaling pathway in astrocytes, which has been identified as essential for diet-induced obesity and hypothalamic inflammation (101). The hypothalamus, as the central hub regulating appetite and energy homeostasis, integrates internal and external signals to maintain metabolic balance. Growing evidence suggests that hypothalamic inflammation may disrupt these processes and propagate inflammatory signals to other CNS regions. In humans, MRI studies reveal hypothalamic gliosis in obesity (102). Murine models show that acute HFD exposure (1 day) elevates hypothalamic IL-6, TNF-α, and microglial activation, with prolonged HFD (3 days) exacerbating neuroinflammation and gliosis. Importantly, hypothalamic microglial inhibition suppresses hyperphagia and weight gain, subsequently improving lipid dysregulation (103). Mechanistically, systemic inflammation and circulating FFAs may target the hypothalamus, potentially inducing local microglial activation and reactive astrogliosis. After 1–4 weeks of HFD feeding, astrocytes in hypothalamic regions secrete inflammatory cytokines that amplify inflammatory cascades (104). These changes may alter hypothalamic circuitry and satiety signaling. Elevated hypothalamic cytokines upregulate suppressor of cytokine signaling 3 (SOCS3), which inhibits insulin/leptin signaling and exacerbates metabolic dysfunction (105). Hypothalamic astrocytes express receptors for various peripheral hormones, including adipokines and insulin (99). Leptin resistance induced by lipid metabolic imbalance impairs astrocytic regulation of feeding behavior, promoting diet-induced obesity. Notably, hypothalamic inflammation often precedes weight gain and peripheral inflammation (102), suggesting it may act as an early responder to metabolic stress.

While the hypothalamus’ role in bridging energy dysmetabolism and depression requires further validation, its early inflammatory response positions it as a potential therapeutic target. We hypothesize that lipid dysregulation-induced hypothalamic inflammation may serve as a mechanistic switch linking metabolic and depressive pathology, warranting investigation as a dual-target strategy.

### 3.5 The lipid dysregulation and synaptic plasticity in the pathogenesis of MDD

Lipid dysregulation disrupts synaptic plasticity-the dynamic adaptability of neuronal connections-constituting a core pathological mechanism underlying cognitive dysfunction in depression (106). Central to this process is the direct disruption of synaptic structure and function caused by lipid dysregulation: specialized membrane microdomains (lipid rafts), whose physical properties depend on precise sphingolipid-cholesterol ratios (107), become rigid when lipid abnormalities reduce unsaturated fatty acid content. This rigidity hinders the functional localization of neurotransmitter receptors (e.g., gamma-aminobutyric acid receptor [GABAR]) and compromises synaptic vesicle fusion with the plasma membrane, inducing “synaptic dysfunction syndrome” (14, 108, 109). These energy-intensive synaptic remodeling processes (e.g., long-term potentiation, LTP) exhibit high sensitivity to alterations in the lipid microenvironment (110).

Lipid dysregulation amplifies synaptic damage by activating microglia and triggering chronic neuroinflammation (111). Under inflammatory conditions, complement components C1q/C3 are overexpressed in the hippocampus and other brain regions, leading to excessive deposition at functional synapses. This aberrant tagging promotes erroneous microglial phagocytosis of synapses, resulting in synaptic loss and cognitive impairment (112, 113). Concurrently, pro-inflammatory cytokines suppress N-methyl-D-aspartate (glutamate) receptor (NMDAR) or α-amino-3-hydroxy-5-methyl-4-isoxazolepropionic acid receptor (AMPAR) expression and phosphorylation via epigenetic mechanisms, weakening glutamatergic signaling and LTP induction (110). Downregulation of astrocytic glutamate transporters exacerbates excitotoxic injury (110), while astrocyte-secreted lipocalin-2 (LCN2) impairs synaptic plasticity by inhibiting neuronal NMDA receptors (114). Critically, brain-derived neurotrophic factor (BDNF), which regulates neuronal survival and synaptic function through tyrosine receptor kinase B (TrkB) receptor signaling, is significantly reduced in depression; inflammatory cytokines disrupt TrkB phosphorylation, worsening BDNF pathway impairment (115, 116).

Lipid dysregulation directly dampens serotonergic (5-HT) neuronal excitability and synaptic transmission efficiency (108), while inflammation-activated indoleamine 2,3-dioxygenase (IDO) diverts tryptophan toward the kynurenine pathway. This metabolic shift reduces 5-HT production, increases neurotoxin creation, and creates a self-perpetuating cycle where inflammation causes tryptophan metabolic imbalance, ultimately leading to neuronal damage that sustains inflammation (117). High-fat and high-sugar diets concomitantly reduce cerebral 5-HT levels and perturb dopamine receptor signaling (118).

In summary, lipid dysregulation can drive the development of depressive disorders by directly disrupting synapses, inducing inflammation, and thereby impairing synaptic plasticity.

### 3.6 Lipid metabolism interacts with the endocrine system to modulate neuroinflammation in MDD

**Figure 3:**
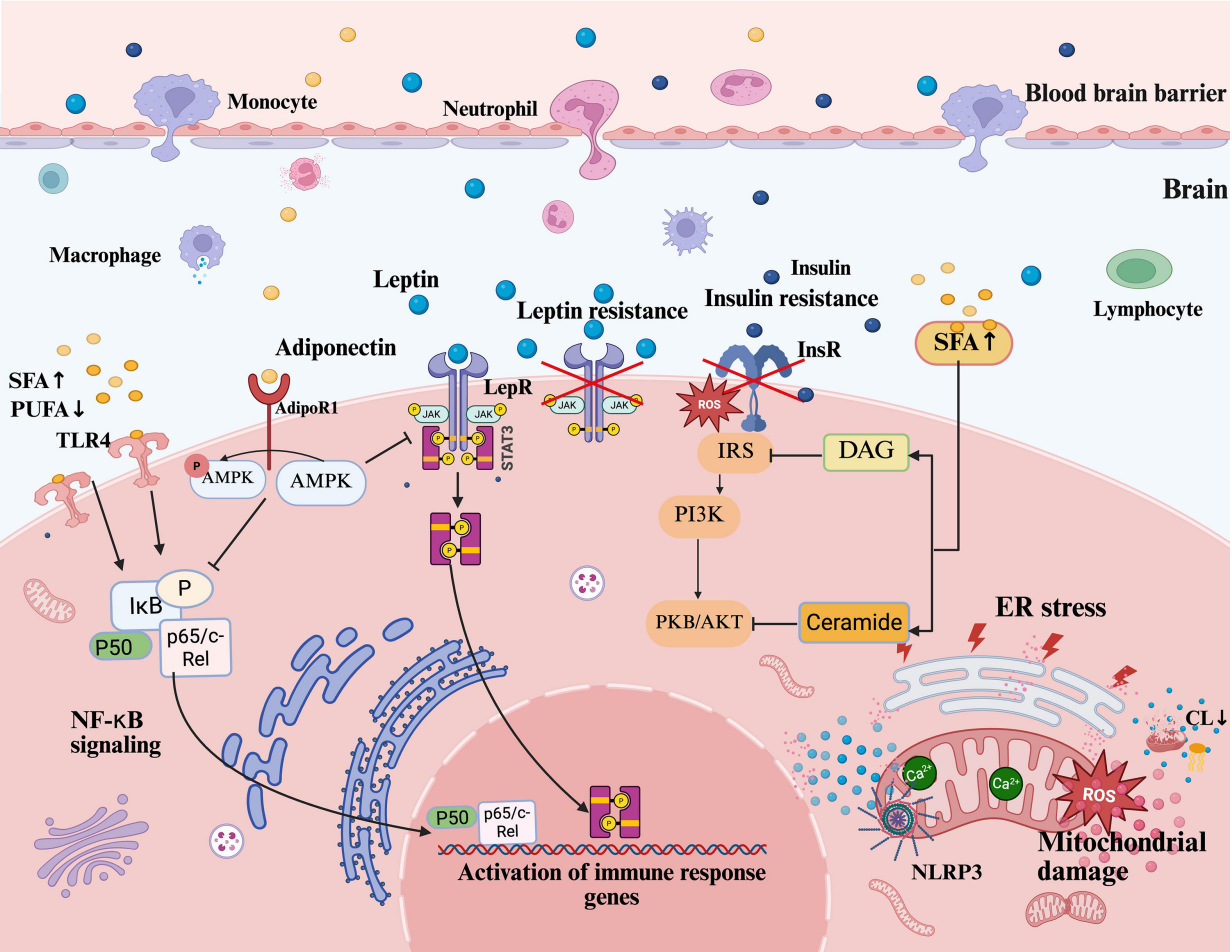
Lipid dysregulation and neuroinflammatory pathways in depression. This figure illustrates how lipid dysregulation drives neuroinflammation and depression via interconnected pathways. Peripheral inflammatory factors and lipid disturbances penetrate the CNS through BBB permeability, promoting neuroinflammation. Excess SFAs activate TLR4, triggering NF-κB signaling (via IκB phosphorylation and p65/c-Rel nuclear translocation) to induce IL-6 and TNF-α. Leptin resistance disrupts JAK2/STAT3 signaling, amplifying inflammation. Dysregulation of lipid metabolism leads to insulin resistance, while SFA-induced DAG and Cer accumulation, which act as signaling lipids disrupting insulin receptor signaling, impair the IRS/PI3K/AKT pathway and exacerbate metabolic dysfunction. Reduced adiponectin diminishes AMPK activation via AdipoR1, weakening anti-inflammatory regulation. Concurrently, lipid dysregulation induces ER stress and mitochondrial dysfunction, elevating ROS and Ca^2+^ imbalance, which activate the NLRP3 inflammasome to release IL-1β. Mitochondrial CL loss exacerbates inflammation. These mechanisms impair synaptic plasticity and hippocampal neurogenesis, driving depressive behaviors, while central neuroinflammation feedback aggravates peripheral lipid dysregulation, sustaining chronicity. CNS, central nervous system; BBB, blood-brain barrier; SFAs, saturated fatty acids; TLR4, toll-like receptor 4; NF-κB, nuclear factor kappa-light-chain-enhancer of activated B cells; IκB, inhibitor of kappa B; IL-6, interleukin-6; TNF-α, tumor necrosis factor alpha; JAK2, Janus kinase 2; STAT3, signal transducer and activator of transcription 3; DAG, diacylglycerol; Cer, ceramide; IRS, insulin receptor substrate; PI3K, phosphatidylinositol 3-kinase; AKT/PKB, protein kinase B; AMPK, AMP-activated protein kinase; AdipoR1, adiponectin receptor 1; ER stress, endoplasmic reticulum stress; ROS, reactive oxygen species; NLRP3, NOD-like receptor family pyrin domain containing 3; IL-1β, interleukin-1 beta; CL, cardiolipin.

Lipid dysregulation forms a vicious cycle with neuroinflammation through hormonal networks, serving as a key hub in depression pathogenesis. HPA axis overactivation triggers cortisol dysregulation, glucocorticoid resistance, and hippocampal atrophy, exacerbating inflammation; insulin resistance disrupts neuronal metabolism and synaptic function, with proinflammatory signals and oxidative stress further impairing insulin signaling pathways and aggravating lipid dysregulation; adipokine imbalance (e.g., leptin/adiponectin ratio disturbance) not only interferes with feeding behavior but also directly mediates immune crosstalk between peripheral and central systems, amplifying neuroinflammation (see **Figure 3**). These interactions translate metabolic dysfunction into neural damage, elucidating depression heterogeneity and offering novel therapeutic perspectives. Below, we focus on the roles of HPA axis dysregulation, insulin resistance, and adipokine disorders in the metabolic-inflammatory cascade.

#### 3.6.1 HPA axis dysregulation: bidirectional stress-inflammation crosstalk

The HPA axis, central to stress responses, regulates glucocorticoid (GC) secretion and is tightly linked to psychiatric pathophysiology (119). Under lipid dysregulation and depression, HPA axis hyperactivity elevates cortisol. Although cortisol itself has anti-inflammatory effects, prolonged high levels of cortisol may induce glucocorticoid resistance, which in turn downregulates GC receptor-mediated transcriptional activity, diminishing its anti-inflammatory efficacy and exacerbating the inflammatory response (120). The hippocampus, as a key brain region for negative feedback regulation of the HPA axis, is atrophied by chronic cortisol excess. Hippocampal damage further exacerbates the dysregulation of the HPA axis, forming a self-reinforcing loop that perpetuates depressive symptoms (2, 10, 120). Hypercortisol affects the kynurenine pathway of tryptophan metabolism by activating tryptophan 2,3-dioxygenase (TDO) activity, leading to serotonin depletion (121), which ultimately triggers depression. This process reveals a strong link between lipid metabolism, inflammation and depression.

Concurrently, lipid dysregulation-driven inflammatory cytokines disrupt HPA axis feedback inhibition (122) and promote cravings for high-fat, high-calorie diets, worsening metabolic imbalance (14, 108). Elevated levels of GC also promote the accumulation of visceral fat, which can exacerbate the peripheral inflammatory response triggered by the lipid dysregulation (2). Notably, atypical depression patients exhibit weight gain, lipid dysregulation, and elevated inflammation despite normal or low GC (123), which suggests that there may be different pathophysiological mechanisms between the depressive subtypes and provides clinical clues to differentiate the depressive subtypes.

Thus, HPA axis dysregulation bidirectionally interacts with lipid metabolism and inflammation, altering dietary behaviors and sustaining a pathological network that exacerbates depression.

#### 3.6.2 Insulin resistance: the intersection of metabolic disorders and neuroinflammation

Clinical studies indicate a significantly higher prevalence of insulin resistance in depressive populations (2). Insulin, as a key metabolic hormone, regulates peripheral energy metabolism and crosses the BBB to modulate feeding behavior, mood, and cognition via CNS actions. Critically, impaired central insulin signaling disrupts neuronal function and synaptic plasticity by altering neurotransmitter channel activity, cerebral cholesterol biosynthesis, and mitochondrial dynamics (124). Brain region-specific expression of insulin-related mRNAs (e.g., in the hypothalamus, hippocampus, and PFC) underscores its neuromodulatory role (125, 126). Astrocyte-specific insulin receptor knockout in mice induces depressive-like behaviors, confirming CNS insulin singling’s pathophysiological relevance (127).

In obesity, reduced brain insulin sensitivity correlates positively with depression and anxiety severity (128). Molecularly, insulin resistance stems from lipid dysregulation: chronic FFA exposure suppresses pancreatic β-cell function and insulin secretion (129). SFAs exacerbate inflammation via c-Jun N-terminal kinase (JNK)/IKKβ/NF-κB activation (JNK/IKKβ/NF-κB signaling pathway), promote diacylglycerol (DAG) accumulation and protein kinase C (PKC) activation (inhibiting insulin receptor substrate (IRS) phosphorylation), and generate Cer that induces Akt dephosphorylation and oxidative stress. These mechanisms synergistically disrupt the IRS/phosphatidylinositol 3-kinase/protein kinase b (IRS/PI3K/Akt) signaling axis (130).

Lipid dysregulation-driven cytokines (e.g., TNF-α, IL-6) further promote insulin resistance (131). Recent studies have revealed that inflammatory factors are central to mediating central insulin resistance: neuroinflammatory states significantly impair insulin signaling in the brain and reduce the antidepressant effects of insulin and its sensitizers (132). Peripheral inflammatory factors interfere with insulin physiology by inhibiting IRS phosphorylation through binding to receptors in the brain in the presence of increased BBB permeability (125). At the organelle level, insulin resistance triggers mitochondrial dysfunction, elevating ROS and oxidative stress (133), which activate NLRP3 inflammasomes and IL-1β release. Impaired insulin signaling also downregulates antioxidant enzymes (134), amplifying oxidative damage and neuroinflammation.

Under conditions of insulin resistance, the inhibitory effect of insulin on lipolysis is attenuated, leading to an elevated release of FFA into the bloodstream, which promotes ectopic lipid deposition. When hypothalamic FFA levels increase, this triggers hypothalamic inflammation and disrupts the homeostatic regulation of energy metabolism by the hypothalamus, further exacerbating lipid dysregulation (99).

#### 3.6.3 Adipokine imbalance (leptin/adiponectin): regulating peripheral-central immunity

Adipose tissue, as an important endocrine organ, secretes adipokines (e.g., leptin, adiponectin) that regulate lipid metabolism and inflammation (135, 136). White adipose tissue (WAT) hyperplasia drives obesity-related chronic inflammation via pro-inflammatory cytokine production. Notably, abnormal secretion of adipokines is significantly associated with the development of psychiatric disorders such as depression. In a state of lipid dysregulation, the levels of pro-inflammatory adipokines (e.g., leptin) are elevated while the secretion of anti-inflammatory adipokines (e.g., adiponectin) is reduced, and this state of imbalance enhances immune cell activation (136, 137). Leptin and adiponectin receptors are widely expressed in the brain, implicating that they may be involved in the pathology of psychiatric disorders through central mechanisms (138). These adipokines modulate neurogenesis, synaptic plasticity, and higher-order functions (e.g., emotion, memory) through metabolic and anti-inflammatory pathways (10, 139).

Leptin stimulates monocyte proliferation, differentiation, and induces pro-inflammatory cytokine production (140, 141). Leptin-deficient mouse model exhibits lymphoid atrophy, immune cell loss, and reduced inflammation (140). Leptin receptor (LepR) is widely expressed on the surface of various types of immune cells, and when leptin binds to the receptor, it activates the Janus kinase 2/signal transducer and activator of transcription 3 (JAK2/STAT3) signaling pathway and promotes the expression of inflammatory factors by inducing homodimerization of LepR (142). Notably, while leptin upregulates the secretion of inflammatory factors such as TNF-α, IL-6, and IL-12, these inflammatory factors in turn can feed back to promote leptin mRNA expression in adipose tissue, forming a pro-inflammatory positive feedback loop (143). Leptin also crosses the BBB, promoting CNS IL-1β release from monocytes and inducing neuroinflammation (144), suggesting that it has a key role in chronic inflammation associated with lipid dysregulation. The hypothalamus, a key target of leptin action, is rich in LepR in the arcuate nucleus. Normally, elevated postprandial serum leptin levels promote pro-opiomelanocortin-expressing neurons (POMC neurons) activation through the JAK2-STAT3 pathway, which suppresses food intake and helps regulate body weight. However, hypothalamic leptin resistance causes dysfunction in this pathway, leading to increased obesity and lipid dysregulation (145).

Clinically, circulating leptin correlates with adiposity (146, 147). In the pathology of depression, leptin levels are often reduced in patients and animal models of chronic stress, whereas obese patients with depression have elevated leptin levels and leptin resistance (148). Chronic stress-induced leptin resistance elevates peripheral inflammation, reversible by leptin-sensitizing interventions (e.g., acupuncture) (149). Elevated leptin levels lead to inflammation, and leptin resistance not only inhibits the appetite suppressant effect of leptin to induce lipid dysregulation but also disrupts the feedback of leptin to increase leptin secretion in adipose tissue, inducing hyperleptinemia and amplifying inflammation. This suggests that abnormal leptin (elevated or resistant) in a state of lipid dysregulation may be involved in the development of depressive disorders through two mechanisms: promoting neuroinflammation or interfering with metabolic signaling.

Adiponectin, another important hormone secreted by WAT, crosses the BBB and is involved in energy homeostasis and appetite regulation, and its receptor is widely expressed in the rodent brain (150, 151). Clinical evidence suggests that peripheral blood levels of adiponectin are significantly lower in depressed patients and that the risk of MDD is significantly increased in a group of men with low adiponectin expression (152–154). Unlike the pro-inflammatory properties of leptin, adiponectin exerts its anti-inflammatory effects by inhibiting macrophage phagocytosis and TNF-α production, and its secretion may be reduced by adipocyte hypertrophy and dysfunction of adipose tissue (155, 156). Mechanistic studies have shown that adiponectin and its receptor AdipoR1 can regulate microglia phenotypic transformation (157). Adiponectin deficiency in mice elevates cortical and hippocampal microglial activation and inflammation (158). Through AdipoR1- amp-activated protein kinase (AMPK) signaling, adiponectin shifts microglia toward the anti-inflammatory M2 phenotype, ameliorating neuroinflammation and depressive behaviors in chronic stress models (157). In addition, its anti-inflammatory effects may involve peroxisome proliferator-activated receptor gamma (PPARγ) or IL-4/signal transducer and activator of transcription 6 (STAT6) pathway modulation (158, 159). Together, the above evidence suggests that adiponectin may exert neuroprotective and pro-neurogenic effects in the hippocampus through mechanisms such as inhibition of neuronal apoptosis and modulation of neuroinflammation.

### 3.7 Lipid dysregulation induces mitochondrial dysfunction, triggering inflammation

Mitochondria, the energy powerhouses of neurons, are critical for maintaining neuronal and axonal bioenergetic homeostasis. Mitochondrial dysfunction strongly correlates with synaptic damage and disrupts monoamine neurotransmitter synthesis, release, and reuptake. Neurons are exquisitely sensitive to mitochondrial dysfunction, and mild hypoplasia can lead to a decline in the efficiency of ATP production, a bioenergetic defect that is strongly associated with cognitive decline in depression and may act as a trigger for inflammation and cell death. Mitochondrial dysfunction markers are observed across multiple brain regions in MDD patients (160).

Lipid dysregulation reduces mitochondrial lipogenesis, accelerates lipolysis, and impairs fatty acid esterification. HFD-induced FFA surges overwhelm mitochondrial β-oxidation capacity, inhibiting respiratory chain enzymes and generating toxic intermediates (e.g., acylcarnitines, long-chain acyl-CoA). Fatty acids accumulated in the vicinity of the mitochondria are susceptible to ROS-induced lipid peroxidation, which poses a lipotoxic threat to the mitochondrial DNA (mtDNA), RNA, and proteins, further compromising function (161). Chronic lipid dysregulation also depletes antioxidant enzymes (e.g., superoxide dismutase [SOD], glutathione peroxidase [GPx]), exacerbating ROS accumulation and mitochondrial damage (162). In MDD, mitochondrial DNA mutations and oxidative injury severity correlate with depressive symptom intensity (163, 164). Mitochondrial membrane permeabilization releases DAMPs, which activate microglial TLR/NF-κB pathways, driving pro-inflammatory cytokine release and neuroinflammation (165). This inflammatory cascade exacerbates mitochondrial dysfunction and ROS overproduction, forming a self-perpetuating cycle.

Lipid dysregulation also disrupts the dynamic balance of mitochondria. Excessive fat intake induces mitochondrial fragmentation into dysfunctional, smaller organelles in WAT of mice, reducing oxidative capacity and lipid utilization, thereby worsening obesity. Dysregulated lipid metabolism promotes adipose tissue aging and inflammation via regulating proteins such as Drp1 and OPA1, which contribute to excessive mitochondrial division and formation of fragmented mitochondria (166, 167). HFD also impairs mitophagy by sequestering microtubule-associated protein 1 light chain 3 (LC3), amplifying NLRP3 inflammasome activation (168). MDD patients with lipid dysregulation exhibit reduced cardiolipin (CL) (169, 170), a mitochondrial inner membrane lipid essential for function and mitophagy. CL depletion destabilizes cytochrome C anchoring, which is a key step in the initiation of apoptotic signaling and may trigger programmed cell death (171). In addition, the absence of CL inhibits the targeting of LC3 to mitochondria, thereby preventing normal mitochondrial autophagic clearance (171, 172). This change not only leads to the accumulation of damaged mitochondria but may amplify the inflammatory response, exacerbating tissue damage and disease progression.

Peripheral and central lipid dysregulation impairs mitochondrial function, alters the brain microenvironment, and initiates neuroinflammation, establishing a vicious cycle that exacerbates depressive pathology.

### 3.8 Lipid dysregulation induces endoplasmic reticulum stress, aggravating inflammatory responses

The ER, a central organelle in lipid synthesis and metabolism, is critical for maintaining lipid distribution and metabolic homeostasis. Emerging evidence implicates ER stress in lipid dysregulation and depression pathogenesis. Chronic depression induces ER functional impairment, leading to ER stress (173, 174). Lipid dysregulation independently triggers ER stress, activating the UPR to restore ER homeostasis. However, sustained stress ultimately promotes inflammation and apoptosis (108, 175).

Membrane fluidity and function are precisely regulated by lipid composition. Imbalanced glycerophospholipid to phosphatidylcholine ratios induce membrane rigidity, disrupting ER protease and transmembrane protein activity, thereby provoking ER stress (175). Lipid dysregulation accumulates bioactive lipids such as SFAs in the ER, causing lipotoxicity, interfering with its function, and triggering stress responses. Chronic excess of SFAs such as palmitate induces irreversible protein palmitoylation, ER stress, and inflammation, while also disrupting ceramide metabolism, sphingolipid homeostasis, ER lipid raft integrity, and calcium (Ca^2+^) storage, ultimately impairing protein folding and triggering cell death (176). The metabolic stress caused by long-term lipid dysregulation may overwhelm the ER with the folding demands of lipid-metabolizing enzymes, worsening misfolded protein buildup, and activating the UPR.

The ER and mitochondria are tightly connected through the mitochondria-associated membrane (MAM), which acts as a central hub of the cellular stress response, leading to depressive behaviors by inducing ER stress and mitochondrial damage (177, 178). Depression and lipid dysregulation induce ER stress, enhancing MAM-mediated ER-mitochondria tethering and Ca^2+^ transfer to mitochondria (179). This causes mitochondrial Ca^2+^ overload, ROS bursts, NLRP3 inflammasome activation, and accelerated depressive-like behaviors. Lipid dysregulation drives neuroinflammatory processes by disrupting mitochondrial and ER homeostasis, and the two can be coupled via MAM to form a metabolic-inflammatory vicious cycle that amplifies the pathological effects of lipid dysregulation.

### 3.9 Potential therapeutic targets for lipid dysregulation and neuroinflammation in the treatment of depression

The established interaction between lipid dysregulation, neuroinflammation, and depressive symptoms highlights the need for new treatment strategies that target these connected pathways. While traditional antidepressants are still the first choice, their inconsistent effectiveness calls for exploring additional approaches specifically aimed at these underlying mechanisms. Potential treatment targets can be categorized based on their main point of intervention.

(1) Targeting Gut-Brain Axis and Microbial Dysbiosis: Given the critical role of gut microbiota in inducing inflammation, disrupting barriers, and signaling via lipids/metabolites (as previously discussed), dietary interventions emerge as a key strategy. Approaches like the Mediterranean diet correct lipid metabolism dysregulation and reduce systemic/neuroinflammation partly by modulating gut microbiota composition and function (180), with limiting SFAs further improving metabolic balance. Direct modulation of the microbiome through probiotics, prebiotics, and FMT aims to restore microbial homeostasis, thereby attenuating inflammation and ameliorating depressive behaviors (181).

(2) Attenuating Neuroinflammation: Building upon the evidence implicating cytokine signaling, microglial activation, and BBB disruption in depression pathophysiology, directly targeting neuroinflammation is crucial. This includes the adjunctive use of anti-inflammatory agents such as NSAIDs, cytokine inhibitors, and statins (which possess pleiotropic anti-inflammatory effects), all showing promise in reducing neuroinflammation and improving depressive symptoms (182, 183). Neuromodulation therapies like transcranial magnetic stimulation (TMS) and deep brain stimulation (DBS) also demonstrate efficacy, in part, through their ability to reduce neuroinflammatory markers and cytokine levels (184). Furthermore, stabilizing the compromised BBB—a key factor facilitating inflammatory crosstalk—using agents like sphingosine-1-phosphate receptor agonists (e.g., fingolimod) represents another targeted approach (185).

(3) Correcting Systemic Metabolic and Neuroendocrine Dysfunction: Addressing peripheral drivers like insulin resistance, adipokine imbalance, and chronic HPA axis hyperactivity (established contributors to neuroinflammation and lipid dysregulation) is vital. Metabolic regulators such as metformin improve insulin sensitivity, alleviating associated depressive symptoms (186, 187). Modulating the hyperactive HPA axis, a central stress response pathway, can be achieved through inhibitors of key signaling nodes like PERK, offering a path to normalize HPA function and mitigate its downstream detrimental effects (188, 189).

Additionally, strategies targeting adipokine dysregulation, such as improving leptin sensitivity via approaches like acupuncture (149), address this specific inflammatory and metabolic link.

(4) Protecting Cellular Organelles and Mitigating Oxidative Stress: Counteracting mitochondrial dysfunction, ER stress, and resultant oxidative damage (key cellular pathologies discussed earlier) provides another therapeutic avenue. Mitochondrial support using targeted antioxidants (e.g., CoQ10, MitoQ) helps mitigate oxidative damage and improve compromised cellular energy metabolism (190, 191). Similarly, relieving ER stress with specific inhibitors (e.g., 4-PBA, TUDCA) aids in restoring proteostasis and cellular homeostasis, countering a significant source of neuroinflammation and neuronal dysfunction (192, 193).

Comprehensive treatment strategies integrating these dietary, pharmacological, and neuromodulatory interventions, each targeting specific nodes within the lipid dysregulation-neuroinflammation-depression axis, offer significant promise for improving depressive symptoms and patient quality of life, particularly where conventional treatments fall short. Future advancements in precision therapies focused on this axis hold potential for more effective solutions in treatment-resistant depression.

## 4 Discussion

Depressive disorders demonstrate strong associations with peripheral lipid dysregulation, reflecting the critical role of lipid homeostasis in brain function (194). As a lipid-dependent organ, the brain utilizes lipids not merely for energy but as signaling molecules essential for neurophysiology. Disrupted peripheral lipid metabolism elevates circulating FFAs, which propagate neuroinflammation via BBB compromise. Concurrently, central lipid dysregulation reciprocally impairs peripheral tissue function, creating a bidirectional pathogenic loop that sustains systemic inflammation and neural damage. This cross-talk exacerbates synaptic dysfunction and depressive progression through mechanisms involving HPA axis hyperactivity, insulin resistance, and adipokine dysregulation. Lipid dysregulation further induces mitochondrial dysfunction and ER stress, triggering oxidative damage and inflammatory cascades that form self-amplifying pathological cycles.

Current diagnostic limitations highlight the need for lipid- and inflammation-focused biomarkers using metabolomics and sequencing technologies (17, 195). Therapeutic strategies should prioritize restoring lipid equilibrium through pharmacological agents, dietary interventions, and lifestyle modifications to suppress neuroinflammatory pathways and alleviate depressive symptoms (157, 194). Integrating these approaches offers promise for developing objective diagnostics and targeted therapies addressing the lipid-neuroinflammation axis in depression.

In addition, this review primarily relies on animal models to elucidate mechanisms; translating these findings to the complex clinical heterogeneity of human MDD requires further validation through human studies. Existing clinical evidence is relatively insufficient, particularly lacking longitudinal studies correlating lipid-neuroinflammatory biomarkers across different depressive subtypes (e.g., melancholic vs. atypical). Furthermore, the efficacy and clinical application of the proposed therapeutic strategies targeting lipid metabolism and inflammation in MDD patient subgroups still require stronger support from clinical trials. The intricate bidirectional interactions between peripheral lipid metabolism, central neuroinflammation, and other systems (e.g., HPA axis, gut microbiota) also complicate the clinical attribution of causality..
